# Complete genome sequence of *Xanthomonas campestris* pv. *campestris* strain NSTU_AG-1 causing black rot disease isolated from infected cabbage leaves in Bangladesh

**DOI:** 10.1128/mra.01484-25

**Published:** 2026-05-18

**Authors:** Arnab Goswami, Maksudur R. Nayem, Pijush K. Jhan, Amena Khatun, A.B.Z. N. Rahman, Mahmudul Hasan, Swagato Dutta, Md T. Islam, Jong-In Park, Mehede H. Rubel

**Affiliations:** 1 Department of Agriculture, Noakhali Science and Technology University 378872, Noakhali, Bangladesh; 2 DNA Solution Ltd., Bir Uttam Quazi Nuruzzaman Sarak, Dhaka, Bangladesh; 3 Department of Microbiology, Noakhali Science and Technology University 378872, Noakhali, Bangladesh; 4 Institute of Biotechnology and Genetic Engineering, Gazipur Agricultural University 198780, Gazipur, Bangladesh; 5 Department of Horticulture, Sunchon National University 65380, Suncheon, Republic of Korea; University of Strathclyde, Glasgow, United Kingdom

**Keywords:** genome sequence, black rot, *Xanthomonas campestris *pv.* campestris*

## Abstract

*Xanthomonas campestris* pv. *campestris* (NSTU_AG-1), the pathogen of black rot disease, was isolated from cabbage in Bangladesh. The genome size was 5,120,760 bp with guanine + cytosine (G + C) content of 64.98% and had no plasmid. The complete genome sequence data of this *Xcc* will help to understand the molecular mechanisms of the pathogen.

## ANNOUNCEMENT

**Fig 1:**
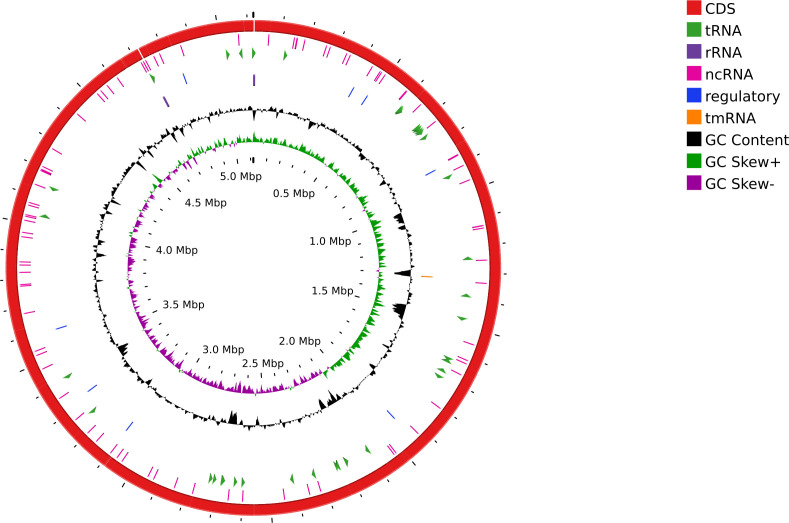
Circular genome map of *Xanthomonas campestris* pv. *campestris* strain NSTU_AG-1 generated using the Proksee server (13). From the outside to the center: circle 1, CDS (red color); circle 2, ncRNA (pink color); circle 3, tRNA (green color); circle 4, regulatory genes (blue color); circle 5, rRNA (violet color); circle 6, tmRNA (deep yellow color); circle 7, GC content (black); and circle 8, GC skew [(G + C)/(G – C)] plotted using a 10-kb window size (purple indicates values below average, while olive represents values above average).

*Xanthomonas campestris* pv. *campestris* (*Xcc*) is a highly destructive bacterial pathogen that causes black rot disease in crucifer crops, leading to significant yield losses (>50%) worldwide, particularly in cabbage (1, 2). Here, we present the complete genome sequence of *Xcc* strain NSTU AG-1 isolated from infected leaves of a cabbage plant (Green Star) showing typical V-shaped chlorotic lesions (3). The leaves were collected during field sampling in Churamonkathi Union (23.22135°N, 89.16882°E), Jessore, Bangladesh. The infected leaf samples were surface sterilized with 3% sodium hypochlorite (NaOCl) for 1 min, followed by several washes with sterile distilled water. Then, only the infected portions of leaf samples containing lesions were cut into small pieces (around 2–3 mm), placed on a King’s medium B base agar plate, and incubated at 30°C for 24–48 h, followed by isolation and purification through subcultures maintaining the same media and conditions (4, 5). The light-yellow colony was picked and inoculated in 10 mL of nutrient yeast broth (NYB) ([2.5% nutrient broth + 0.5% yeast extract] wt./vol.) at 160 rpm and 30°C for DNA extraction (6). Subsequently, the liquid culture broth was sent to DNA Solution, Ltd., Dhaka, Bangladesh, maintaining 4°C, where the whole-genome sequencing was performed. DNA was extracted from bacteria using a QIAamp DNA Mini Kit (QIAGEN, Germany) and quantified with a Qubit 4 fluorometer (Thermo Fisher Scientific, USA). Using the native barcoding kit (SQK-NBD-114.24), the library was prepared from extracted bacterial DNA without shearing or size selection. The DNA was ligated with ONT barcode and adapter and sequenced in the Oxford Nanopore MinION Mk1C Platform utilizing the FLO-MIN114 (R10.4.1) flow cell (Oxford Nanopore Technologies, UK). Data were collected using MINKNOW.1.11.5 (Oxford Nanopore Technologies, UK). Using Guppy ver. 6.3.2 (Oxford Nanopore Technologies) in high accuracy mode, MinION Mk1C sequencing data (FAST 5 format) were basecalled to generate pass reads (FASTQ format) with a mean quality score > 9. The number of raw reads was 17,260, and the read length of *N*_50_ was 14,077. The adaptor and barcode sequences were trimmed using Porechop v0.2.4 (https://github.com/rrwick/Porechop), while Nanofilt v2.8.0 was used to trim low-quality bases (q < 10) (7). The quality-trimmed reads were assembled into one contig using Flye Version 2.9.5 through *de novo* assembly method (8). Flye provided a circular assembled genome where the genome length was 5,120,760 bp with a coverage of 60×. CheckM v1.2.4 quantified genome contamination and completeness by the *Xanthomonas campestris* CheckM marker set (9). Genome annotation of *Xcc* strain NSTU AG-1 was performed using Prokaryotic Genome Annotation Pipeline (PGAP) v6.10 (10). The genomic features were summarized and represented in Table 1 and in the circular map (Fig. 1). DNA:DNA hybridization (89.2%; Formula 2: d4) and average nucleotide identity (ANI) (98.75%) between the assembled genome and the closest taxon, *Xcc* strain ATCC 33913 (National Center for Biotechnology Information [NCBI] accession no. NC_003902.1), using GGDC 3.0 and ANI calculator (https://www.ezbiocloud.net/tools/ani), respectively, confirmed it as *Xcc* (11, 12). These genomic data of *Xcc* strain NSTU_AG-1 from Bangladesh will help researchers understand this pathogen and build sustainable disease management techniques.

**TABLE 1:** Genomic features of *Xanthomonas campestris* pv. *campestris* strain NSTU_AG-1

Features	Value
Genome length	5,120,760 bp
Contigs	1
G + C content	64.98%
Plasmids	0
*N*_50_	5,120,760
Genome completeness	98.25%
Contamination	1.11%
tRNA	54
rRNA	6
Total coding sequences (CDS)	4,366
Protein-coding CDS	4,162
Non-protein-coding CDS	204
Genes	4,512

## Data Availability

The complete genome sequences have been deposited in GenBank under accession number CP199799.1 (chromosome). The BioProject accession number is PRJNA1321718, and the BioSample accession number is SAMN51222686. Raw reads are available at the NCBI SRA under accession number SRX30953418.
